# Clinical manifestation, epidemiology, genetic basis, potential molecular targets, and current treatment of polycystic liver disease

**DOI:** 10.1186/s13023-024-03187-w

**Published:** 2024-04-26

**Authors:** Amir Ali Mahboobipour, Moein Ala, Javad Safdari Lord, Arash Yaghoobi

**Affiliations:** 1 Tracheal Diseases Research Center, National Research Institute of Tuberculosis and Lung Diseases (NRITLD), Shahid Beheshti University of Medical Sciences, Tehran, Iran; 2 Experimental Medicine Research Center, School of Medicine, Tehran University of Medical Sciences (TUMS), Tehran, Iran; 3 Department of Medical Genetics, School of Medicine, Tehran University of Medical Sciences, Tehran, Iran; 4 School of Biological Science, Institute for Research in Fundamental Sciences (IPM), Tehran, Iran

**Keywords:** ADPKD, ARPKD, ADPLD, Cystogenesis, Liver cyst, Polycystic liver disease, Mutation

## Abstract

Polycystic liver disease (PLD) is a rare condition observed in three genetic diseases, including autosomal dominant polycystic liver disease (ADPLD), autosomal dominant polycystic kidney disease (ADPKD), and autosomal recessive polycystic kidney disease (ARPKD). PLD usually does not impair liver function, and advanced PLD becomes symptomatic when the enlarged liver compresses adjacent organs or increases intra-abdominal pressure. Currently, the diagnosis of PLD is mainly based on imaging, and genetic testing is not required except for complex cases. Besides, genetic testing may help predict patients’ prognosis, classify patients for genetic intervention, and conduct early treatment. Although the underlying genetic causes and mechanisms are not fully understood, previous studies refer to primary ciliopathy or impaired ciliogenesis as the main culprit. Primarily, PLD occurs due to defective ciliogenesis and ineffective endoplasmic reticulum quality control. Specifically, loss of function mutations of genes that are directly involved in ciliogenesis, such as *Pkd1, Pkd2, Pkhd1,* and *Dzip1l,* can lead to both hepatic and renal cystogenesis in ADPKD and ARPKD. In addition, loss of function mutations of genes that are involved in endoplasmic reticulum quality control and protein folding, trafficking, and maturation, such as *PRKCSH*, *Sec63, ALG8*, *ALG9*, *GANAB*, and *SEC61B,* can impair the production and function of polycystin1 (PC1) and polycystin 2 (PC2) or facilitate their degradation and indirectly promote isolated hepatic cystogenesis or concurrent hepatic and renal cystogenesis. Recently, it was shown that mutations of *LRP5,* which impairs canonical Wnt signaling, can lead to hepatic cystogenesis. PLD is currently treated by somatostatin analogs, percutaneous intervention, surgical fenestration, resection, and liver transplantation. In addition, based on the underlying molecular mechanisms and signaling pathways, several investigational treatments have been used in preclinical studies, some of which have shown promising results. This review discusses the clinical manifestation, complications, prevalence, genetic basis, and treatment of PLD and explains the investigational methods of treatment and future research direction, which can be beneficial for researchers and clinicians interested in PLD.

**Supplementary Information:**

The online version contains supplementary material available at 10.1186/s13023-024-03187-w.

## Introduction

PLD is a shared presentation of several genetic diseases such as ADPKD, ARPKD, and ADPLD [1–4]. PLD is generally a rare medical condition mainly observed together with polycystic kidney disease (PKD) rather than alone [5–7]. Unlike PKD, which can finally progress to end-stage renal disease (ESRD), PLD does not impair liver function, but instead, liver enlargement physically compresses the adjacent organs and increases intra-abdominal mechanical pressure, which can cause most of the symptoms and necessitates treatment in symptomatic cases [8, 9]. Although PLD remains asymptomatic in a considerable proportion of patients [9].

Currently, PLD is diagnosed based on imaging modalities such as ultrasonography, computed tomography (CT) scan, and magnetic resonance imaging (MRI). Identification of more than 20 hepatic cysts commonly confirms the diagnosis of PLD [7, 9, 10]. Due to the widespread use of abdominal imaging for various purposes, asymptomatic PLD or early-stage PLD is usually diagnosed as an incidental finding in many cases [7]. Pathological assessment can show many fluid-filled cysts whose lining is covered by cholangiocytes [9, 10]. In addition to the vast genetic heterogeneity among cases, already-known genetic variants do not explain all cases and the responsible genes in a substantial group of patients still need to be discovered [11].

In this review, we dissect the clinical manifestation, complications, prevalence, genetic basis, and treatment of PLD. In addition, we discuss the investigational methods of treatment and future research direction based on the underlying molecular mechanisms. As this article comprehensively discusses all dimensions of the topic, it can be helpful for researchers, scientists, and clinicians who wish to know the latest findings regarding PLD.

## Clinical presentation and epidemiological characteristics

### Clinical presentation and epidemiological characteristics of ADPKD

PKD is a genetic disorder that causes the growth of fluid-filled cysts in the kidneys and damages the surrounding tissues [12, 13]. ADPKD is the most common form of PKD and the most frequent hereditary kidney disease, which finally progresses to ESRD [14]. A meta-analysis of 8 epidemiological studies revealed that the prevalence of ADPKD is approximately 2.7 per 10,000 individuals [15]. ADPKD can present with hypertension, pain, hematuria, urinary tract infection, proteinuria, liver cysts, intracranial aneurysms, heart valve insufficiency, and mitral valve prolapse [14]. Although this disease is inherited monogenetically, it is phenotypically and genetically heterogeneous [12, 13]. Progressive renal fibrosis in ADPKD is often associated with extrarenal abnormalities such as cystogenesis in the liver, seminal vesicle, pancreas, and arachnoid membrane, abdominal herniation, intracranial aneurysms, and cardiac abnormalities [2, 12, 13]. Hepatic cysts are the most common extrarenal manifestations of ADPKD, and the incidence of hepatic cysts among patients with ADPKD was shown to gradually increase with aging [1, 2]. Among 129 patients with ADPKD in one study, 62.8% of participants developed PLD [16]. Despite renal cysts, hepatic cysts do not develop in utero and mainly manifest after puberty [1]. In addition, age was an independent predictor of hepatic cysts in patients with ADPKD [1, 17]. Moreover, female gender, number of pregnancies, severity of renal cystic disease, and renal functional impairment were positively associated with the progression of PLD in patients with ADPKD [1, 17]. Another study comprising 241 patients with ADPKD and 119 patients with ADPLD indicated that female patients with ADPKD had larger height-adjusted total liver volume (TLV) compared with female patients with ADPLD [18]. Surprisingly, the study reported that among patients with ADPKD, younger females (≤ 51 years) had greater liver volumes than older females (> 51 years), reminding the importance of female sex hormones in the development of liver cysts [18].

Consistent with the effect of female gender and pregnancy in hepatic cyst growth [1, 17], it was found that estrogen receptor and insulin-like growth factor 1 (IGF1) receptor were markedly upregulated in hepatic cyst epithelium, and 17β-estradiol and IGF1 significantly promoted liver cyst-derived epithelial cell proliferation [19].

Comparing the clinical characteristics of 19 patients with isolated ADPLD and 34 patients with ADPKD revealed that [20]: 1) development of liver cysts was significantly correlated with female gender in both ADPLD and ADPKD; 2) Patients with ADPLD had greater numbers and larger sizes of liver cysts but experienced fewer morbidities; 3) Liver cyst decompressions were significantly more frequent among patients with ADPLD, and serious hepatic complications necessitating liver transplantation were more common in ADPKD [20].

### Clinical presentation and epidemiological characteristics of ARPKD

Autosomal recessive polycystic kidney disease (ARPKD) is a less common form of PKD. Its prevalence is estimated to be 1 in 20,000 live births [21]. ARPKD usually manifests during pregnancy or childhood, leading to premature death [22]. Of 50 patients with ARPKD, 24% were diagnosed before birth and 66% were diagnosed before 1 year of age, with hypertension as the most common symptom [22]. ARPKD is characterized by the development of multiple cysts in the kidneys and liver, as well as other complications such as pulmonary hypoplasia and hypertension [22, 23]. Hepatic complications are also frequently detected in patients with ARPKD, including hepatic fibrosis, hepatosplenomegaly, portal hypertension, cholangitis, variceal bleeding, ascites, hepatic and bile duct cysts, and hepatic fibrosis [3, 22, 23]. Particularly, cholangitis, portal hypertension, and subsequent variceal bleeding, splenomegaly, and thrombocytopenia are the main and most severe hepatic complications of ARPKD [24]. Liver cysts have been observed in the ultrasonography of 23.1% of patients with ARPKD [3]. Among 32 patients with ARPKD and pathogenic variants of the *Pkhd1* gene, one-third exhibited prenatal anomalies, and five died within the first year of life due to respiratory failure [25]. Another cross-sectional study, which analyzed 49 patients with ARPKD and a mean age of 21.4 ± 3.3 years, reported that fourteen (31%) patients underwent kidney transplantation and six patients (13%) underwent liver transplantation or both liver and kidney transplantation [26].

### Clinical presentation and epidemiological characteristics of ADPLD

The incidence of ADPLD seems to be 1.01 per 100,000 person-years and most cases are detected between 30 and 50 years of age [7, 27]. ADPLD is characterized by abnormal liver enlargement, which physically compresses the adjacent organs [27]. Patients with isolated ADPLD mainly present with abdominal pain, abdominal distension, dyspepsia, and dyspnea, and less than 20% of patients may remain asymptomatic [9]. Compared with individuals with a negative or indeterminate diagnosis of ADPLD, those with ADPLD were shown to have slightly higher serum levels of alkaline phosphatase, gamma-glutamyl transferase, and total bilirubin and lower serum levels of total cholesterol and triglyceride [27]. It has also been observed that female patients with ADPLD develop more advanced liver cysts compared with male patients [27]. The hepatic cysts in patients with ADPLD originate from the proliferating biliary microhamartomas and peribiliary glands [27]. In addition to cyst hemorrhage, rupture, and infection, the growing hepatic cysts may compress the neighboring organs and cause serious complications, such as portal vein obstruction, common bile duct obstruction, and inferior vena cava occlusion, that often necessitate urgent medical intervention [8, 9]. PLD is also accompanied by increased mechanical pressure on the abdominal wall, which considerably elevates the risk of abdominal herniation [28]. A study comprising 484 patients with PLD reported that 40.1% of patients developed abdominal hernia, particularly umbilical hernia [28]. Therefore, the management of ADPLD mainly aims to reduce liver volume or prevent liver enlargement. However, these compressive symptoms due to liver enlargement are the main symptoms in ADPLD, they can all be expected in ADPKD and ARPKD since PLD is a common manifestation of all of these diseases.

In addition, several classification systems, such as Schnelldorfer classification (Supplementary Table 1), Gigot classification (Supplementary Table 2), and Qian classification, have been developed to categorize disease severity and symptomatic phase in PLD [29, 30]. Schnelldorfer and Gigot classifications consider the size and number of cysts and normal liver parenchyma [29, 30]. Schnelldorfer classification also considers portal vein or hepatic vein occlusion for categorization and relates symptom burden to the number of affected liver segments [30]. However, Qian classification simply categorizes patients with PLD into 5 grades based on the number of liver cysts and the presence of symptoms [27].

## Genetic basis

### Genetic basis of ADPKD

ADPKD is caused by mutations in either *Pkd1* or *Pkd2* gene, which encode polycystin-1 (PC1) and polycystin-2 (PC2), respectively. PC1 and PC2 are involved in the development and maintenance of kidney cells, and their mutations can lead to the growth of fluid-filled cysts [31].

Mutations of *Pkd1* gene on chromosome 16p13.3 and *Pkd2* gene on chromosome 4q22.1 account for almost 80% and 15% of ADPKD cases. The remaining 5–10% of ADPKD cases are not genetically determined or occur due to rare mutations at other loci [31]. Some cases of PKD can be explained by mutations in at least one of the endoplasmic reticulum protein-encoding genes. The loss of any of these genes, such as *GANAB, DNAJB11,* and *ALG9,* results in the production of non-functional PC1 [31–33].

*GANAB*, also known as *Pkd3*, encodes the alpha subunit of glucosidase II. The main function of glucosidase II is to promote protein folding by catalyzing the hydrolysis of glucose residues of immature glycoproteins. *GANAB* mutation can disrupt protein maturation and cell surface localization of PC1 and PC2 [34]. Studies have shown that *GANAB* variants cause mild polycystic kidney and liver cysts in most patients [35]. DNAJB11 is a co-factor of binding immunoglobulin protein (BiP), which is a major chaperone in the endoplasmic reticulum and regulates the folding, trafficking, and degradation of secreted and membrane proteins [36]. DNAJB11 deletion was shown to impair PC1 maturation and trafficking [36]. Likewise, heterozygous loss of function mutation of the *ALG9* gene, which encodes an enzyme needed for adding specific mannose molecules to produce N-glycan precursors in the endoplasmic reticulum, can impair PC1 maturation and lead to the development of kidney and liver cysts [33].

**Fig. 1:**
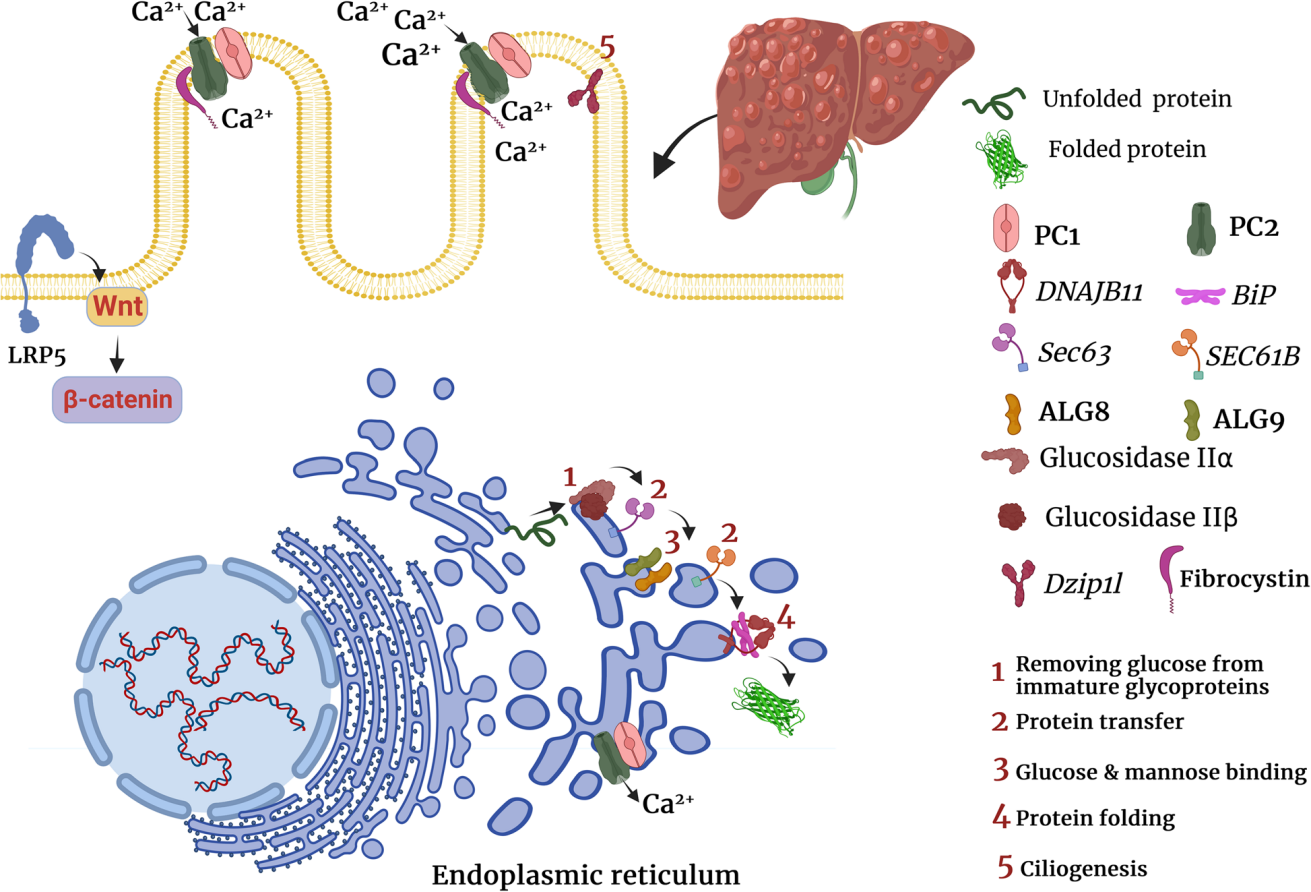
The role of PLD-causing genes in cholangiocytes. As shown in the figure, PLD-causing genes are primarily involved in ciliogenesis and quality control of protein folding, transport, and maturation in the endoplasmic reticulum

*Pkd1* or *Pkd2* deletion promotes renal tubular cell proliferation, which was shown to be associated with higher intracellular concentrations of Ca^2+37^. PC2 mainly localizes on the endoplasmic reticulum, primary cilia, and plasma membrane, acts as a cation channel, and forms the PC1-PC2 complex in a 1:3 ratio [38, 39]. PC2 acts as an ion channel on the plasma membrane and allows a small but detectable Ca^2+^ influx in renal primary cilia; therefore, mutated PC2 is deemed to decrease intracellular Ca^2+^ concentration [40]. PC2 acts as a potassium channel in the endoplasmic reticulum to facilitate potassium–calcium counterion exchange for inositol trisphosphate–mediated endoplasmic reticulum Ca^2+^ release [41]. PC2 also directly functions as a calcium-activated, high-conductance ER channel mediating Ca^2+^ release from the endoplasmic reticulum [42], and *Pkd2* knockout impairs Ca^2+^ release from the endoplasmic reticulum in kidney cells [41]. In addition, PC1 was shown to decrease Ca^2+^ leak from the endoplasmic reticulum and increase endoplasmic reticulum Ca^2+^ uptake [43, 44]. It has been hypothesized that PC1 may physically block cation transfer by PC2 [39, 45]. Membrane depolarization and increased intraciliary Ca^2+^ concentration both can activate monovalent cation transfer by PC2 ^39^. In addition, PC2 is needed for PC1 localization in the cilia, and PC2 deletion not only promotes cystogenesis but also inhibits ciliary localization of PC1 [46]. Furthermore, Yao et al*.* reported that *Pkd1* knockout can enhance PC2 expression by upregulating GRP94, an endoplasmic reticulum chaperone [47]. Enhancing *Pkd2* expression in *Pkd1*-mutant cells may improve PC1 trafficking or promote the formation of heteromeric PC1-PC2 protein complexes (Table 1 and Fig. 1) [48].

**Table 1:** Genes involved in the pathogenesis of PLD

Gene	Role	Disease	Reference
*Pkd1*	Its product, PC1, forms a complex with PC2 and regulates its function	ADPKD	[48]
*Pkd2*	Its product, PC2, acts as a cation channel and regulates the concentration of Ca^2+^ in endoplasmic reticulum and in intracellular space	ADPKD	[37][39],
*GANAB*	Its product, glucosidase IIα hydrolyzes glucose residues of immature glycoproteins and promotes protein folding	ADPKD	[34][35],
*DNAJB11*	It is a co-factor for BiP, which is a major chaperone in the endoplasmic reticulum. DNAJB11 deletion was shown to impair PC1 maturation and trafficking	ADPKD	[36]
*ALG9*	It encodes an enzyme that adds specific mannose molecules to produce N-glycan precursors in the endoplasmic reticulum. Its mutation impairs PC1 maturation	ADPKD	[33]
*Pkhd1*	Its product, fibrocystin, forms a complex with PC2 on the plasma membrane and controls Ca^2+^ transfer	ADPKD	[25][59],
*Dzip1l*	It is necessary for ciliary bud formation and encodes a ciliary transition zone protein that is responsible for ciliary membrane translocation of PC1 and PC2	ADPKD	[60][61],
*PRKCSH*	Its product, glucosidase IIβ, binds the C-terminal domain of PC2 and inhibits Herp-mediated ubiquitination and subsequent degradation of PC2 and PC1	ADPLD	[62][63],
*Sec63*	It is mainly involved in protein transport in the endoplasmic reticulum	ADPLD	[62][63],[64],
*ALG8*	As a glucosyltransferase family, it is involved in the endoplasmic reticulum quality control and is needed for the maturation and trafficking of PC1	ADPLD	[32]
*SEC61B*	It is involved in in endoplasmic reticulum quality control and is needed for the maturation and trafficking of PC1	ADPLD	[32]
*LRP5*	It is involved in the regulation of canonical Wnt signaling pathway, and its mutation can promotes the expression of several proliferative genes	ADPLD and ADPKD	[65, 66]

*Abbreviations:* polycystic liver disease (*PLD*), polycystin 1 (*PC1*), polycystin 2 (*PC2*), binding immunoglobulin protein (*BiP*), autosomal dominant polycystic liver disease (*ADPLD*), autosomal dominant polycystic kidney disease (*ADPKD*), and autosomal recessive polycystic kidney disease (*ARPKD*)

The morphological assessment of hepatic cyst epithelium in patients with ADPKD illuminated that small (< 1 cm) hepatic cysts had normal epithelium, medium-sized (1–3 cm) hepatic cysts had rare or shortened cilia, and large (> 3 cm) hepatic cysts lacked both primary cilia and microvilli [19]. Normally, primary cilia are assumed to promote cellular quiescence and delay cell cycle progression to the S or M phase [49]. In addition, ciliary disassembly was shown to induce cell-cycle reentry [49]. Consistently, it was shown that decreased ciliogenesis in cancer cells enhances their proliferative capacity and promotes their invasive behavior [50].

The classical hypothesis for cyst formation claims that in addition to a germline inactivating mutation in one allele of the *Pkd* gene, there is somatic inactivation (referred to as the second hit) in another allele, causing the complete loss of polycystin expression. However, recent studies claimed that the function of the *Pkd* gene has a threshold for cystogenesis [51, 52]. Based on this hypothesis, complete loss of *Pkd1* function is not required, and partial malfunctioning of *Pkd1* is enough to induce cystogenesis [53]. Consistently, many individuals with ADPKD still have residual PC1 expression because they carry missense rather than inactivating mutations [54]. Thus, promoting the expression of the normal *Pkd1* allele may improve ADPKD even in the presence of an abnormal allele. The type of mutation not only determines the development and penetrance of ADPKD but also explains the severity of cystogenesis [16]. A study with 129 participants with ADPKD revealed that mutation position and mutation type (truncating mutation: nonsense, frameshift, and splicing mutation; or non-truncating mutation: substitution) can affect the severity of hepatic cystogenesis, and patients with *PKD1* nonsense mutations exhibit more severe hepatic cystogenesis [16]. Furthermore, in this study, ADPKD patients with *Pkd1* nonsense mutation located closer to the 5ʹ end of *Pkd1* gene were more likely to have a maximum diameter index value of hepatic cyst ≥ 6 cm [16].

### Genetic basis of ARPKD

ARPKD is caused by mutations in the polycystic kidney and hepatic disease 1 (*Pkhd1*) gene, which encodes fibrocystin/polyductin. Different variants of the *Pkhd1* gene (missense and truncating mutations) cause most cases of ARPKD. The mRNA of *Pkhd1* is alternatively spliced to generate multiple transcripts [55, 56]. *Pkhd1* knockout was shown to promote cholangiocyte proliferation in vitro [57]*.* Furthermore, it was found that *Pkhd1* knockout induces connective tissue growth factor (CTGF) production by cholangiocytes, which can induce hepatic fibrosis [57]. Similar to PC1, fibrocystin forms a complex with PC2 on the plasma membrane and participates in Ca^2+^ transfer [58]. Previously, it was found that the COOH terminal of fibrocystin interacts with the NH2 terminal of PC2. The lack of fibrocystin decreased PC2 expression, but *Pkd2* deletion did not alter fibrocystin expression ^59^. These findings suggest that fibrocystin binds to PC2 and maintains its normal levels, thereby preventing cystogenesis (Table 1 and Fig. 1) [59].

In another study, it was shown that children with clinically moderate ARPKD had a mutation in the *Dzip1l* gene [60]. Similar to the *Pkhd1* gene, the *Dzip1l* gene is involved in ciliogenesis [61]. *Dzip1l* deletion downregulated ciliogenesis or led to the formation of dysmorphic cilia in mice [61]*. Dzip1l* gene encodes a ciliary transition zone protein that is responsible for ciliary membrane translocation of PC1 and PC2 (Table 1 and Fig. 1) [60].

### Genetic basis of ADPLD

Mutations in *PRKCSH* or *Sec63* genes have been implicated in the development of ADPLD [62]. *PRKCSH* or *Sec63* mutations are found in approximately 40% of patients with isolated ADPLD [9]. *PRKCSH* and *Sec63* genes encode glucosidase IIβ and SEC63p, respectively, and are involved in endoplasmic reticulum quality control [62]. They are responsible for carbohydrate processing and folding and translocation of newly synthesized glycoproteins [62]. As a chaperone-like molecule, glucosidase II binds to the C-terminal domain of PC2 and inhibits Herp-mediated ubiquitination and subsequent degradation of PC2 [62]. Likewise, *PRKCSH* or *Sec63* deletion was shown to impair normal PC1 folding and accelerate its ubiquitination and proteasomal degradation [63]. *Sec63* conducts the post-translational transport of proteins in the endoplasmic reticulum (Table 1 and Fig. 1) [64]. Consistently, proteasome inhibition by MG132 and carfilzomib, two proteasome inhibitors, markedly upregulated PC1 and promoted cyst-lining cell apoptosis [63].

Using whole-exome sequencing data from 102 unrelated patients, Choi et al*.* demonstrated that heterozygous loss of function mutations in 3 additional genes, *ALG8*, *GANAB*, and *SEC61B*, are also linked to ADPLD [32]. Using in vitro experiments, they also indicated that similar to *PRKCSH* and *SEC63*, *ALG8*, *GANAB*, and *SEC61B* are related to protein biogenesis pathway in the endoplasmic reticulum and loss of function mutation of each one of these genes results in defective maturation and trafficking of PC1 (Table 1 and Fig. 1) [32].

A recent study has shown that heterozygous mutations of the low-density lipoprotein receptor-related protein 5 (*LRP5*) gene, particularly p.R1188W variant, can lead to ADPLD; however, another study reported that some variants of *LRP5*, such as rs724159825, can also lead to ADPKD [65, 66]. Mechanistically, *LRP5* mutations were shown to impair canonical signaling of Wnt3α and promote the expression of several proliferative genes such as adenomatous polyposis coli (APC), glycogen synthase kinase 3β (GSK3β), and leucine-rich repeat-containing G-protein-coupled receptor 5 (LGR5), transcription factor v-myc avian myelocytomatosis viral oncogene homolog (c-Myc), and cyclin D1 (Table 1 and Fig. 1) [66].

### Is genetic testing helpful in the diagnosis and treatment of PLD?

Currently, genetic screening is not widely used to confirm ADPKD, ARPKD, and ADPLD as their imaging characteristics and clinical presentations are distinct and there are few differential diagnoses [67]. On the other hand, already known disease-causing genetic mutations include a wide spectrum and still do not explain a considerable proportion of cases, particularly in ADPLD [68]. In addition, it has been shown that the affected gene or the type of mutation cannot significantly alter the phenotype of PLD [67]. Therefore, current guidelines do not recommend routine genetic testing for PLD [67].

However, genetic testing is not necessary to confirm ADPKD, ARPKD, and ADPLD or enough to rule out these diseases; it may help categorize patients and potentially identify those eligible for future modalities of genetic intervention. Furthermore, a recent study reported that genetic confirmation can predict the risk of hospitalization in both isolated and non-isolated PLD [69]. Specifically, the study indicated that mutation carriers were significantly younger when waitlisting for liver transplantation and first hospitalization compared to patients without genetic diagnosis; however, current imaging classifications could not differentiate between severe and moderate courses [69].

Genetic testing can also be helpful when patients come with atypical presentations, which mimic other diseases and make diagnosis complex for clinicians [68]. In addition, genetic testing is the last resort when patients present with clinical symptoms or complications, but their cyst number in imaging still does not satisfy the diagnostic criteria for ADPLD or ADPKD [68]. On the other hand, with recent findings and future advances toward the pharmacological and genetic interventions for ADPLD, ADPKD, and ARPKD, genetic testing can allow early diagnosis and management of these diseases. Early diagnosis and management can considerably improve patients’ outcome and prevent serious complications [68]. Therefore, future studies may define new applications for genetic testing of PLD.

### Potential molecular targets for treating PLD

*Pkd1* and *Pkd2* mutations have been linked to deregulated activation of proliferative signaling pathways. Indeed, decreased intracellular Ca^2+^ concentration following impaired function of PC2 is believed to be responsible for activating proliferative pathways ^70^. Intracellular Ca^2+^ depletion can activate adenylyl cyclase 5, which in turn upregulates intracellular cyclic adenosine monophosphate (cAMP) levels [70]. Increased cAMP can subsequently overactivate protein kinase A (PKA)/Ras/extracellular signal-regulated kinases (ERK)/hypoxia-inducible factor α (HIF-α) pathway, promote vascular endothelial growth factor A (VEGF-A) expression, and enhance angiogenesis for cholangiocyte proliferation [71, 72]. Consistently, adenylyl cyclase 5 inhibition and knockout both significantly reduced hepatic cystogenesis in *Pkd* knockout mice [70]. Likewise, VEGF receptor inhibition was shown to inhibit liver cyst growth in pkd2 (WS25/ −) mice [73], and serum levels of VEGF were positively correlated with total cyst volume but negatively correlated with creatinine clearance in patients with ADPKD [74]. Moreover, PKA inhibition in liver cyst epithelial cells decreased VEGF expression and ERK1/2 activation [71]. ERK inhibition also reduced the proliferation of liver cyst epithelial cells [71].

Janus kinase (JAK)/signal transducer and activator of transcription (STAT) pathway is also aberrantly activated in ADPKD and contributes to epithelial cell proliferation [75, 76]. It was shown that JAK2 expression strongly increases in ADPKD and JAK2 blockade reduces cyst growth. JAK2 is a key kinase that most likely contributes to cyst growth by activating STAT as a transcription factor [77].

Similar to the JAK/STAT signaling pathway, dysregulated mechanistic target of rapamycin (mTOR), Wnt, and Hippo signaling pathways have also been implicated in the pathogenesis of ADPKD. It was shown that the mTOR pathway is abnormally activated in cyst-forming epithelial cells in patients with ADPKD and in the mice model of ADPKD [78]. Rapamycin, an mTOR inhibitor, was shown to effectively suppress cystogenesis in two mouse models of PKD. Moreover, treatment with rapamycin markedly decreased native polycystic kidney size in patients with ADPKD who received kidney transplants [78].

Similarly, it has been indicated the lack of PC2 can overactivate the Wnt/β-catenin pathway in murine embryonic fibroblasts, renal epithelia, and isolated collecting duct cells [79]. In addition, inhibition of the Wnt/β-catenin pathway prevented renal cyst formation and prolonged survival in a mice model of ADPKD [79]. Similarly, non-canonical Wnt/planar cell polarity (PCP) pathway has been implicated in the proliferative response after *Pkhd1* mutation in ARPKD [80]. Wnt can also bind to the extracellular domain of PC1, thereby inducing PC2-dependent Ca^2+^ influx in epithelial cells [81]. Pathogenic mutations in *Pkd1* and *Pkd2* were shown to abrogate PC1-PC2 complex formation, reduce cell surface localization of PC1, and hinder PC2 activation by Wnt molecule ^81^. Besides, mutations in several PLD-causing genes, such as *LRP5*, *Sec63*, and *Pkhd1,* were shown to impair Wnt signaling pathway, which makes it interesting for further investigation [66, 80, 82].

Previously, it has been reported that overactivation of Hippo/Yes-associated protein (YAP) and their transcriptional target four-jointed (*Fjx1*) is a major driver of cystogenesis in ADPKD [83]. Consistently, it was shown that simultaneous knockout of *Fjx1* decelerates renal fibrosis, alleviates renal inflammation, and preserves renal function in mice with *Pkd1* deletion; however*, Fjx1* knockout did not markedly inhibit cyst formation [84].

As PC1-PC2 complex deficiency leads to decreased intracellular Ca^2+^ concentration, activation of transient receptor potential vanilloid (Trpv4), a calcium-entry channel in cholangiocytes, has been proposed as a therapeutic option ^86^. In-vitro experiments showed that Trpv4 activation increases intracellular Ca^2+^ concentration and decreases cholangiocyte proliferation and cyst growth in 3-dimensional culture [85]. In vivo, Trpv4 activation significantly reduced renal cystic area and non-significantly reduced liver cysts [85]. Similarly, it was found that Trpv4 activation downregulates cAMP levels and decelerates the progression of ARPKD in rats [86].

Using tissues from patients with ADPLD and in vivo and in vitro experiments, it was shown that increased HDAC6-mediated ubiquitination and deregulated autophagy of ciliogenic proteins such as ADP-ribosylation factor-like protein 3 (ARL3) and ADP-ribosylation factor-like protein 13B (ARL13B) in cholangiocytes promote hepatic cystogenesis [87, 88]. In addition, inhibition of autophagy was shown to promote ciliary localization of ARL3 and ARL13B, recover cholangiocyte ciliogenesis, and inhibit uncontrolled proliferation of cholangiocytes [87, 88]. Interestingly, it was indicated that increased autophagic removal of miR-345 potentiates hepatic cystogenesis in PLD [89]. miR-345 is a non-coding RNA that targets and downregulates cell cycle and proliferation-related genes such as cell division cycle 25A (*CDC25A*), cyclin-dependent kinase 6, E2F transcription factor 2, and proliferating cell nuclear antigen [89]. These findings point out the importance of autophagy as a therapeutic target in PLD.

Inhibition of protein SUMOylation with S-adenosylmethionine or protein NEDDylation with pevonedistat, as post-translational events, hindered hepatic cystogenesis in the experimental model of PLD [90, 91]. Inhibition of autophagy by hydroxychloroquine also suppressed the proliferation of PLD cholangiocytes in vitro and decreased hepatic cystogenesis in a rat model of ADPKD [88]. Pioglitazone and telmisartan can act as peroxisome proliferator-activated receptor γ (PPAR-γ) agonists. Activating PPAR-γ signaling pathway by pioglitazone or telmisartan reduced liver size and decreased PLD progression in the rat model of ARPKD [92, 93]. Previously, it was found that CDC25A is overexpressed in the cholangiocytes of patients with PLD or PKD and in rats with PKD [94]. Furthermore, *Cdc25A*^±^ *Pkhd1*^del2/del2^ mice, with nearly 50% decreased Cdc25A expression, had 33% reduction in liver weight compared with Pkhd1^del2/del2^ mice ^95^. Consistently, a CDC25A inhibitor like vitamin K3 or PM-20 diminished liver and kidney cystogenesis in *Pkd2*^WS25/−^ mice model ^95^.

Discovery of new disease-causing mutations and identification of the signaling pathways that mediate cystogenesis can provide new therapeutic targets for PLD.

### Treatment

PLD usually does not impair liver function. Therefore, the latest European Association for the Study of the Liver (EASL) guideline limited the treatment indication to symptomatic patients whose symptoms are attributable to cysts and liver enlargement [67].

Treatment options available for PLD can be classified into three categories: pharmacological treatment (especially somatostatin analogs), radiological or percutaneous intervention, and surgery [67]. Since liver size is a prognostic marker in PLD, the efficacy of therapeutic strategies is usually measured by changes in TLV. For this purpose, CT or MRI is the gold standard for liver volume measurement in patients with PLD [95].

EASL decision-making flowchart suggests somatostatin analogs for PLD patients with numerous scattered small-to-medium-sized cysts. Surgical resection is the treatment of choice if these cysts are clustered in a few liver segments. Aspiration sclerotherapy and cyst fenestration are recommended or a single giant cyst and multiple superficial large cysts, respectively. Finally, liver transplantation may be the last solution for massive PLD that severely affects the quality of life [67]. Here, we discuss the treatment strategies and the latest evidence.

### Pharmacological treatment

Cyclic adenosine monophosphate (cAMP) is a principal regulator of cholangiocyte proliferation and fluid secretion. Octreotide, as a somatostatin analog, binds to the somatostatin receptor, reduces cAMP levels in cholangiocytes and serum, and prevents cyst growth [4]. Several randomized controlled trials (RCTs) investigated the efficacy of pharmacotherapy, especially long-acting analogs of somatostatin, in patients with PLD (Table 2) [96–104]. They demonstrated that somatostatin analogs can reduce TLV compared to placebo [96–101].

**Table 2:** Completed randomized controlled trials on pharmacotherapy regimens for polycystic liver disease

Author (Trial Registry Code)	Study Design	Included Patients	Arms	Primary Outcome and Safety (Arm 1 in comparison with Arm 2)
Keimpema et al*.* 2009 [96] (NCT00565097)	Randomized double-blind parallel assignment	54 patients with PLD (ADPLD and ADPKD) from the Netherlands and Belgium	1) Lanreotide-LAR 120 mg SC every 28 days for 24 weeks 2) Placebo	Imaging modality: CT TLV: -2.9% vs. + 1.6%, *p*-value < 0.01 No severe adverse events related to the intervention
Hogan et al*.* 2010 [97] (NCT00426153)	Randomized double-blind parallel assignment	42 patients with PLD (ADPLD and ADPKD) from the USA	1) Octreotide-LAR 40 mg IM every 28 days for one year 2) Placebo	Imaging modality: MRI (CT in three patients) TLV: -4.95% vs. + 0.92%, *p*-value < 0.05 No serious adverse events related to the intervention
Caroli et al*.* 2010 [98] (Not registered) (Post-hoc analysis)	Randomized double-blind cross-over assignment	12 patients with PLD (ADPKD) from Italy	1) Octreotide-LAR 40 mg IM every 28 days for six months 2) Placebo	Imaging modality: CT TLV change: -71 ± 57 mL vs. + 14 ± 85 mL, *p*-value < 0.05
Pisani et al*.* 2016 [99] (NCT02119052) (Post-hoc analysis)	Randomized single-blind parallel assignment	27 patients with PLD (ADPKD) from Italy	1) Octreotide-LAR 40 mg IM every 28 days for three years 2) Placebo	Imaging modality: MRI TLV change: -7.8% vs. + 6.1%, *p*-value < 0.01 Treatment-related serious adverse events: one asymptomatic cholelithiasis and one acute cholecystitis
van Aerts et al*.* 2019[100] (NCT01616927) (Post-hoc analysis)	Randomized open-label parallel assignment	175 patients with PLD (ADPKD) from the Netherlands	1) Lanreotide-LAR 120 mg SC every 28 days for 120 weeks 2) Standard care	Imaging modality: MRI h-TLV: -1.99% vs. + 3.92%, p-value < 0.001 Serious adverse events: 30.1% vs. 12.2%
Hogan et al*.* 2020[101] (NCT01670110)	Randomized double-blind parallel assignment	48 patients with PLD (ADPLD and ADPKD) from USA	1) Pasireotide-LAR 60 mg IM every 28 days for one year 2) Placebo	Imaging modality: MRI TLV: -3% vs. + 6%, *p*-value < 0.001 Serious adverse events: 12% vs. 13%, *p*-value = 0.91
Wijnands et al*.* 2018 [102] (NCT02048319)	Randomized double-blind parallel assignment	34 patients who underwent aspiration sclerotherapy of a symptomatic dominant liver cyst (23 patients had PLD) from the Netherlands	1) Pasireotide-LAR 60 mg IM two weeks before and two weeks after the aspiration sclerotherapy 2) Placebo	Imaging modality: ultrasonography Median cyst diameter reduction: 23.6% vs 21.8%, *p*-value = 0.96 Serious adverse events: 12% vs. 12%
D'Agnolo et al*.* 2016 [103] (NCT02021110)	Randomized open-label parallel assignment	34 patients with PLD (ADPLD and ADPKD) from the Netherlands and Spain	1) UDCA oral in a dose of 15–20 mg/kg/day for 24 weeks 2) Standard care	Imaging modality: CT TLV change: + 4.6% vs. + 3.1%, *p*-value = 0.49 No serious adverse events related to the intervention
Chrispijn et al*.* 2013 [104] (NCT01157858)	Randomized open-label parallel assignment	44 patients with PLD (ADPLD and ADPKD) from the Netherlands	1) Octreotide-LAR 40 mg IM every four weeks + everolimus 2.5 mg oral daily for 48 weeks 2) Octreotide-LAR 40 mg IM every four weeks for 48 weeks	Imaging modality: CT TLV change: -3.8% vs. -3.5%, *p*-value = 0.73 Serious adverse events: 14% vs. 9%

*Abbreviations: PLD* polycystic liver disease, *ADPLD* isolated autosomal dominant polycystic liver disease, *ADPKD* autosomal dominant polycystic kidney disease, *h-TLV* height-adjusted total liver volume, *IM* intramuscular, *SC* subcutaneous, *LAR* long-acting release, *TLV* total liver volume

In a phase three RCT conducted by van Aerts et al*.,* 175 PLD patients (as an external manifestation of ADPKD) with at least 2000 mL liver volume were included. The intervention group received 120 mg of lanreotide every 28 days via subcutaneous injection. After 120 weeks, compared with the control group, height-adjusted TLV decreased by 5.91% (95% CI: -9.18 to -2.63; *p*-value < 0.001); however, the symptom severity score did not significantly differ between the two groups. The main serious adverse event, probably related to lanreotide, was liver cyst infection in 6.5% of patients in the intervention group. They concluded that long-term treatment with lanreotide can reduce liver growth in this setting [100]. Moreover, this benefit could be seen in short-term therapy with lanreotide [96]. In another study, changing the lanreotide dose from 90 to 120 mg in non-responders, which was administered subcutaneously every four weeks for one year, stopped the increase in TLV. Thus, the efficacy of lanreotide may be dose-dependent [105].

A recently published systematic review and meta-analysis on RCTs (mainly administering octreotide 40 mg or lanreotide 120 mg every 28 days with at least a six-month follow-up) confirmed the effectiveness of somatostatin analogs for PLD treatment [96–101, 106]. They are associated with a lower liver growth rate (mean difference = -6.37%, 95% CI: -7.90 to -4.84; *p*-value < 0.001) compared to the control group. This effect is also seen for total kidney volume (mean difference = -3.66%, 95% CI: -5.35 to -1.97; *p*-value < 0.001). However, they do not significantly affect eGFR decline (mean difference = -0.96 mL/min./1.73 m2, 95% CI: -2.38 to 0.46; *p*-value = 0.19). Regarding adverse events, biliary complications, gastrointestinal symptoms, and cyst infection occurred more frequently in the somatostatin group than in the control group [106].

Some studies showed that cessation of treatment (drug holiday) with somatostatin analogs can lead to the recurrence of cyst growth [107, 108]. Meanwhile, retreatment with somatostatin analogs after a drug holiday was as effective as the first cycle of treatment regarding TLV reduction. Therefore, intermittent doses of somatostatin analogs can be considered in a subset of patients [108].

Other drugs also showed a promising potential to reduce liver volume in animal studies, but their efficacy was disappointing in clinical trials [103, 104]. In polycystic rats, ursodeoxycholic acid (UDCA) has been shown to stop hepatic cystogenesis by increasing intracellular calcium levels [109]. However, 24 weeks of treatment with oral UDCA (15–20 mg/kg/day) did not decrease TLV in patients with PLD (*p*-value = 0.49). Despite this fact, post hoc analysis showed that in patients with ADPKD, UDCA decreased liver cyst volume growth [103]. Thus, further studies are needed to evaluate the efficacy of UDCA in PLD. mTOR inhibitors such as everolimus and sirolimus, best known for their roles in cancer therapy and kidney transplant, demonstrated their effectiveness in the preclinical setting [78, 110–112]. Nevertheless, clinical trials did not support their efficacy for PLD. An add-on trial showed that the combination of everolimus and octreotide is not superior to octreotide alone in reducing TLV (-3.8% vs. -3.5% respectively, *p*-value = 0.73) [104].

As mentioned previously, the number of pregnancies and female gender are associated with the number and size of hepatic cysts in ADPKD [17]. Estrogen stimulates cholangiocyte proliferation by activating the extracellular signal-regulated kinase (ERK) signaling pathway [113]. A case report mentioned that in a 59-year-old woman with breast cancer and ADPLD, treatment with tamoxifen, 20 mg once daily for five years, markedly decreased the volume of liver cysts from 311 to 22 mL [114]. In addition, each year of exposure to estrogen-containing oral contraceptives was associated with 1.45% higher height-adjusted TLV among premenopausal women with PLD [115]. Moreover, postmenopausal estrogen therapy in women with ADPKD was significantly associated with a selective increase in total liver volume but not with kidney volume [116]. Furthermore, an ongoing RCT in the Netherlands evaluates the efficacy of a gonadotropin-releasing hormone (GnRH) agonist in pre-menopausal women with PLD (NCT05478083).

Ultimately, gene therapy may be the future landscape for PLD treatment. PC1 is a large membrane glycoprotein, which is too huge to be modified by gene therapy. However, a recently published animal study concluded that only a tiny piece of this protein could be enough to prevent the disease. A transgenic expression of 200 amino acid-long fragment of PC1 dramatically suppressed kidney cystogenesis in a *Pkd1*-knockout murine model. This finding opens a new insight into the gene therapy of ADPKD [117].

### Percutaneous or radiological intervention

Cyst aspiration and sclerosis are recommended for PLD patients with a symptomatic large cyst (> 5 cm) [95]. In this method, the interventionist aspirates cystic fluid and then injects sclerosing agents such as ethanol, tetracycline, or minocycline to destroy the cyst wall epithelium [118–120]. A systematic review including 526 patients showed that this procedure reduced cyst size by 76%-100% and eliminated the symptoms of PLD in 56%-100% of patients. However, not all patients had PLD, and the recurrence rate was not reported [121]. Besides, PLD patients usually have multiple cysts, and this method does not apply to most PLD patients.

Transcatheter arterial embolization (TAE) is another percutaneous procedure that utilizes an embolic agent to occlude the supplying arteries [122]. In a retrospective cohort study with 244 PLD patients, TAE significantly reduced liver volume by 9.2% after one year of the procedure [123]. Moreover, Yan et al*.* observed an approximately 15% decrease in TLV in 13 patients with PLD 6–12 months following TAE [124]. Meanwhile, Yang et al*.* reported that among 18 PLD patients who underwent TAE, the failure rate was around 70% [125]. It is why the EASL guideline has not recommended TAE for PLD patients [67].

### Surgical management

For superficial large hepatic cysts, cyst fenestration can be considered in symptomatic PLD patients [95]. This technique consists of cyst fluid aspiration and surgical deroofing, mostly through laparoscopic surgery. Compared to aspiration sclerotherapy, the main advantage of this method is that multiple cysts can be treated in one session [126]. In a meta-analysis of 62 studies on patients with or without PLD, symptoms alleviated in 90% of patients after laparoscopic fenestration; however, subgroup analysis showed that symptom recurrence rate and the complication rate are as high as 34% and 29% among patients with PLD, respectively [127]. Additionally, an ongoing RCT aims to compare the efficacy of aspiration sclerotherapy with laparoscopic fenestration in patients with large symptomatic hepatic cysts (NCT05500157).

When the cysts are limited to a few hepatic segments, hepatic resection can be a therapeutic approach for PLD. However, hepatectomy should only be performed in severely symptomatic patients who are not suitable candidates for liver transplantation [67]. Although it can remarkably reduce liver volume and relieve symptoms, the morbidity rate is up to 50% [126]. Among 186 patients with PLD, the mortality rate of surgical treatment was 2.7%, and 21% of patients experienced major complications after dual therapy with hepatectomy and fenestration [128]. Furthermore, hepatectomy can complicate future liver transplantation since it causes abdominal adhesion [129].

Finally, the only cure for patients with PLD is liver transplantation. Liver transplantation has a better prognosis in PLD than in chronic liver failure or hepatocellular carcinoma (5-year patient survival rate 85%) [130]. However, liver transplantation is not commonly used for patients with PLD since the number of liver donors is limited, and PLD is not a medical emergency and has a low mortality rate [95]. One of the available allocation systems is the model of end-stage liver disease (MELD) score. However, this model has been validated for cirrhosis. In the PLD setting, liver transplantation is considered for patients with extensive PLD whose quality of life is severely affected by the liver disease, or who experience serious complications, such as recurrent cyst infections, portal hypertension, variceal bleeding, and severe malnutrition, and when other interventions fail or are not suitable. Moreover, in patients with creatinine clearance less than 30 ml/min surgeons can consider combined liver and kidney transplantation [67]. One of the reasons for hepatorenal transplantation in patients with PLD/PKD is malnutrition and cachexia due to the compressive effect of the liver on the stomach. Malnutrition is a dangerous complication of PLD that can be seen in severe cases, especially in cases where there is concurrent renal failure [67, 131]. In the study by Coquillard and colleagues, the 5-year survival rate of patients with PLD/PKD who underwent hepatorenal transplantation was 90%. In contrast, the 5-year survival rate of PLD patients who underwent liver transplantation was 77%, and that of patients who underwent hepatorenal transplantation for other reasons was 67%. The authors speculate that the difference in survival between the two groups PLD/PKD and PLD was caused by the difference in transplant indication, as the transplant indication for patients with PLD/PKD was mostly poor renal function [131].

## Conclusion

PLD is caused by different genes and can be observed alone or in combination with PKD. Primarily, PKD occurs due to defective ciliogenesis and ineffective endoplasmic reticulum quality control of ciliogenic proteins. Currently, PLD is mainly diagnosed by imaging and treated by surgical fenestration, resection, and liver transplantation in advanced stages. Future genetic interventions based on recent findings about the genetic basis of PLD may open a new chapter for research and bring hope to patients. An increasing number of studies are now uncovering the genetic basis and subsequent signaling pathways and mechanisms that are responsible for hepatic cystogenesis. Identification of the underlying genetic mutations and subsequent alterations in cellular signaling pathways can help develop new therapeutic options and decrease the need for liver transplantation. In addition, clinical trials have shown that pharmacological intervention might be helpful to some extent, and previous in vivo studies have indicated the involvement of several signaling pathways in the development of PLD. By targeting these signaling pathways, more satisfactory results may be obtained in clinical trials.

### Supplementary Information

**Supplementary Material 1.**

## Data Availability

Not applicable.
