# Supplementary material for: Clinical manifestation, epidemiology, genetic basis, potential molecular targets, and current treatment of polycystic liver disease
Source: Orphanet J Rare Dis. 2024 Apr 26;19:175. doi: 10.1186/s13023-024-03187-w (PMC11055360; doi:10.1186/s13023-024-03187-w)
Supplement: Supplementary file 1 — Supplementary Material 1. [file 13023_2024_3187_MOESM1_ESM.docx]

**Supplementary** **Table 1. Schnelldorfer classification for the severity of PLD.**

|  | **Symptoms** | **Cyst characteristics** | **Areas of relative normal liver parenchyma** | **Isosectoral portal vein or hepatic vein occlusion of preserved sector** |
| --- | --- | --- | --- | --- |
| **Type A** | Absent or mild | Any | Any | Any |
| **Type B** | Moderate or severe | Limited Number with large cysts | > 2 sectors | Absent |
| **Type C** | Severe (or moderate) | Any | > 1 sector | Absent |
| **Type D** | Severe (or moderate) | Any | > 1 sector | Present |

The liver segments are left lateral segment, left medial segment, right anterior segment, and right posterior segment.

**Supplementary** **Table 2. Gigot classification for the severity of PLD.**

|  | **Number of cysts** | **Cyst size** | **Remaining areas of non-cystic liver parenchyma** |
| --- | --- | --- | --- |
| **Gigot type I** | < 10 | Large (> 10 cm) | Large |
| **Gigot type II** | Multiple | Small, medium | Large |
| **Gigot type III** | Multiple | Small, medium | Few |
